# Hibernating or not hibernating? Brown bears’ response to a mismatch between environmental natural cues and captive management, and its welfare implications

**DOI:** 10.1371/journal.pone.0306537

**Published:** 2024-07-31

**Authors:** Paolo Dori, Isabella Anastasio, Elisabetta Macchi, Isabella Manenti, Maik Hones, Monica Carosi

**Affiliations:** 1 Department of Science, Roma Tre University, Rome, Italy; 2 Department of Veterinary Science, University of Turin, Turin, Italy; 3 Zoosafari di Fasano, Fasano, Italy; University of Veterinary Medicine Vienna: Veterinarmedizinische Universitat Wien, AUSTRIA

## Abstract

In wild brown bears, likely factors triggering hibernation response to harsh environmental conditions are temperature, photoperiod, and food resources availability. In fact, constantly fed captive brown bears are described as skipping hibernation being active all year-round. Is the hibernation response so flexible and subordinate to contingencies, or else is an adaptation that, if dismissed, may negatively impact on bear well-being? This study investigates the potential hibernation response in captive brown bears under unvaried management conditions using an integrative approach simultaneously analyzing multiple animal-based variables together with environmental covariates. Data from a mid-latitude zoo revealed distinct behavioral, fecal glucocorticoids, and body condition score seasonal fluctuations, resembling natural hibernation cycles, despite constant food access. Environmental variables like photoperiod and visitor numbers significantly influenced activity levels. Bears exhibited behaviors indicative of hyperphagia and fall transition, such as appetitive feeding and denning behaviors. Hormonal analyses revealed high fecal cortisol metabolites levels during hyperphagia, suggesting physiological responses to seasonal changes. Findings underscore the importance of environmental cues and food availability in shaping zoo bear behavior and physiology. Considering that the hibernating vs. non-hibernating description might represent an oversimplification, management strategies should deal with captive bear potential need to freely express their adaptive predispositions by accommodating their natural behaviors, such as providing denning spots and adjusting diet composition as soon as typical hyperphagic and predenning behaviors emerge, ultimately enhancing their well-being.

## Introduction

Brown bears (*Ursus arctos*) are a widespread species inhabiting a great variety of habitats over portions of three continents, from Western Europe eastwards through northern Asia to the western areas of North America [1]. Remarkable seasonal behavioral and physiological changes are characteristic of this species (e.g., activity levels [2–4], body temperature [4, 5], body mass [6, 7], heart rate [4, 8], cardiac structure and function [9], thyroid hormones [10]) and altogether traditionally classify this species as hibernating.

Observational evidence is best described by the seasonality shown in activity levels, which in fact, are described as high during spring and summer, then decreasing during fall, and at their lowest during winter months, with these yearly crucial phases usually recognized as: hyperphagia, fall transition, hibernation and hypophagia, respectively (e.g., [11–13]). During hyperphagia, bears intensely search for high calorie food in order to accumulate fat for the winter fasting (e.g., like berries in Sweden [14], like hard mast in Italy [13] and Spain [15]). At fall transition, which may generally last one or two weeks, bears spend time near the den site, with digging and nest material collection as typical pre-denning behaviors (e.g., [14]). During hibernation, wild bears usually exhibit continuous dormancy for months without eating, drinking, defecating, or urinating [16]. Finally, hypophagia corresponds to those months following den emergence after hibernation, characterized by a low feeding activity and preceding a phase of progressively high activity levels which includes the mating season (e.g., [17]). In sum, bears go through a physiological dormancy aimed at energy savings during ecologically demanding periods, such as the combination of both low temperatures and food scarcity (i.e., winter time in the Northern emisphere; e.g., [18–20]). Main environmental triggers of hibernation are reported to be temperature, photoperiod and food availability [3, 4, 12, 20–23]. Captive management of such a species, genetically programmed and physiologically adapted to hibernate (reviewed in [24]), and whose metabolism is strongly affected by circadian rhythms (e.g., [25]), might require a specific attention to the best practices accommodating bears needs.

Within the animal welfare study framework, the variety of perspectives found in the literature could be summarized by three main approaches to welfare, each emphasizing a specific aspect, namely: the biological functioning (i.e., promoting health, growth and reproduction), the affective state (i.e., minimizing suffering and promoting positive emotional experiences), and the natural living (i.e., granting the opportunity to express natural behaviors and adaptations) [26–28]. Within their role in conservation, research, and education [29], modern zoos represent an exceptional context in which the combination of all three welfare approaches are becoming increasingly important. A literature survey has shown a variety of zoo/research center practices in bear management, ranging from not focusing on bear hibernation adaptation, keeping an unvaried bear management year-round and just letting bears flexibly respond to local environmental conditions (e.g., [30, 31]); to actively supporting a manifest tendency to enter hibernation, by providing the right resources to do so (e.g., nutritionally varied food, denning spots and bedding material, e.g., [12]); to finally purposely inducing hibernation as a standard management by artificially mimicking environmental conditions (i.e., over-feeding during hyperphagia coupled with a feeding break in winter, and manipulating ambient temperature and lighting, e.g., [32, 33]). Interestingly, not only the impact of each of these management practices on bear welfare has not been evaluated yet, but also the entangled relation between hibernation mechanisms and environmental triggers in wild and captive brown bears is still under investigation.

In the wild, according to latitude, a great variability in the timing of den entry and exit (broadly ranging from October to May, [18]) is reported, with earlier den entry and longer hibernation period occurring in the northern than in the southern areas [21, 34].

Manchi and Swenson [21] suggested the longer denning periods in northern latitudes being likely the results of harsher climates and limited food availability, whereas spring emergence being regulated by increasing daylength and warmer temperatures. Evans and colleagues [4] observed that the reduction in body temperature during den entry is driven by a reduction in ambient temperature, meaning that delayed den entry could be a consequence of warmer climates. These results are partially consistent with research conducted on a long-term dataset (69 years) which showed how den entry and exit are affected and strongly associated with respectively decreasing and increasing ambient temperature [22]. On the other hand, Evans and colleagues [4] observed that den exit, differently from den entry, was not dependent on a set ambient temperature. In fact, the narrow range of body temperatures reported among bears on the day of exit suggested that den emergence occurred when they reached a specific set point after several months of thermoregulatory processes aimed at restoring euthermia. Therefore, hibernation in brown bears seems to be mostly triggered by environmental cues however terminated due to physiological cues [4].

The role of photoperiod in affecting brown bear activity levels shows conflicting results. Although some identified it as one of the main environmental factors involved in regulating the activity cycle (e.g., [4, 12]), photoperiod had no apparent impact on bear physiology since it did not correlate either with variation in body temperature or with decrease in heart rate [4]. Also, McLellan & McLellan [3] found that the average amount of daylight per week (i.e., photoperiod) was not influencing activity levels in 19 wild brown bears throughout the year. Recently, Thiel and collaborators [20] found that photoperiod was influential on both activity and physiology (body temperature and heart rate) of bears during their active phase, whereas physiology rhythms were slowed down during hibernation, when perception of light cues is limited.Other studies found that brown bears are likely more sensitive to food availability, as opposed to daylength and temperature [3, 12], highlighting the significance of food in determining the activity levels and patterns. In fact, bears can adapt the timing of denning to food availability during hyperphagia [23] and hibernation takes place to reduce energy loss in winter when food is unavailable [35]. Also, when food is abundant in wintertime, whether naturally or else human provided, denning can be disrupted, such as in Kodiak Island (where some Kodiak bear did not den at all [36]) and Slovenia [37].

Yearly physiological changes also include a seasonal pattern of serum cortisol concentration [38–40]. Cortisol is best known for inducing the anabolic process of gluconeogenesis that increases the availability of blood glucose when the body needs energy, as in the hypothalamic-pituitary-adrenal axis mediated stress response (e.g., [41]). Nevertheless, it also regulates lipid metabolism by controlling the expression of a variety of both lipogenic and lipolytic genes in several tissues [42]. In fact, bear body mass undergoes seasonal variation by increasing before hibernation (body mass gain during hyperphagia) and decreasing during hibernation, until the hypophagic period (Eurasian brown bears [7]) with glucocorticoids likely regulating both processes. Sergiel and colleagues [38] found higher glucocorticoid metabolite levels during the hyperphagic compared to the hypophagic period and since cortisol is also indirectly implied in regulating appetitive behavior and food intake (e.g., [43]), its increase during hyperphagia may be explained by the need to gain body fat (i.e., lipogenic effect) in preparation for winter. Actually, cortisol seasonal pattern was disrupted in wild bears when artificially fed year-round [38]. Higher glucocorticoids were even found during hibernation compared to the active period in wild bears (black bears [44], brown bears [45]) when increasing lipolysis is necessary to provide energy from fat during prolonged winter fasting [46].

Also, body mass gain and loss show flexibility in diverse environmental conditions, being more pronounced in Northern than Southern Europe probably due to the harsher conditions in the north [7]. In fact, brown bears show an extensive behavioral, ecological and even physiological flexibility as an adaptation to cope with highly variable and diverse environmental conditions, depending not only on latitude but also on local seasonality [12, 47–49].

Studies on bears whose management was not focused on hibernation (i.e., bears kept yearly on a regular feeding schedule) reported only a slight seasonal variability in behavior [31, 50], physiology [30, 50], and body mass (mentioned in [30] [Unpublished]), labelling bears as non-hibernating. Despite this, similarities with the wild hibernating conspecifics were mentioned at the physiological level, namely for insulin resistance (American black bears [30]), and creatinine, both increasing during winter season [50]. Insulin resistance, in particular, characterizes an independent seasonal change in metabolism that could explain body mass fluctuations, despite captive bears being fed year-round (American black bears [30]). In captive non-hibernating bears no data for cortisol seasonal patterns are available to our knowledge. On the contrary, in captive brown bears whose hibernation was induced, cortisol concentration was higher during hibernation compared to hyperphagic period [39] and during autumn (hyperphagic period) compared to preceding summer [40], as also reported for their wild hibernating conspecifics [38, 45].

### Aim of the study

Brown bears living in unmanaged captivity (hibernation-wise) in zoos located at temperate latitudes, are likely to undergo conflicting environmental signals due to the lack of correspondence between the seasonality of climate/photoperiod and the non-seasonality of the availability/variety of nutritional food values and appropriate spots/materials for denning. Research on captive brown bears in some cases dismisses the scenario of a slight seasonal variability in behavior, and/or physiology, and/or body mass throughout the seasons, as one typical of non-hibernating bears, in sum exceedingly simplifying the hibernating response to a “yes/no issue”. Nevertheless, the potential bear hibernation response to unmanaged captive conditions, as characterized by a multimodal dimension (i.e., behavior, physiology, and morphology) used to study multiple subjects, has yet to be thoroughly investigated. We believe this might contribute to better evaluate the necessity of providing bears with a whole environment that is temporally changing with congruity, in relation to bear welfare. We took advantage of a captive setting (a) located in a mid-latitude geographic area (i.e., characterized by seasonality in both photoperiod and climate), (b) that provided bears with free access to constant food resources, and (c) that neither prompted, assisted, or encouraged hibernation, in order to evaluate whether and to what extent the mismatch between the seasonality of climatic triggers and the non-seasonality especially of food resource availability, might disrupt the natural hibernation response in brown bears.

By using an integrated animal-based approach we checked for behavioral, hormonal, and morphological indicators which could disclose seasonal trends and in turn describe in more detail the animals’ potential hibernation response to rather constant captive management conditions. In particular, we tested bear inactivity/activity, fecal cortisol metabolites, and body condition during potential seasonal phases described for wild bears, namely the hyperphagic, fall transition, hibernation and hypophagic phases. We used statistical models to include as covariates the main climatic variables (such as photoperiod and ambient temperature) resulting from the literature as triggers in prompting and regulating wild bear’s response to environmental seasonality.

Captivity, however, may offer an additional confounding scenario, due to the ambivalent effects of zoo visitors on both behavior and physiology, as related to the animal stress response in either positive or negative way (described in a variety of mammal species [51, 52]). In brown bears, available studies report an uncertain visitor influence on behavior with either negative effects (increasing frequencies of stereotypy and vigilance, affecting both active and inactive behaviors [53]) or no effects (no differences in rates of stereotypy or social behavior when related to daily visitor attendance [54]). In addition, whereas visitor presence correlated with increase in cortisol concentration in some non-bear mammal species (e.g., Asian elephant, *Elephas maximus*, black rhino, *Diceros bicornis*, clouded leopard, *Neofelis nebulosa*, Mexican wolf, *Canis lupus baileyi* [55]), no hormonal response was found in brown bears (e.g., [56]). Due to conflicting literature about possible role of visitor presence in affecting stress response, and due to the typical seasonal pattern of visitor attendance in zoos, number of visitors was also considered as an environmental covariate. We used a statistical approach which allowed us to simultaneously compare different not mutually exclusive models and to analyze the response variables in relation to singular and combined covariates. Results will be discussed within the framework of animal welfare impact and management.

## Materials and methods

### Subjects, housing and husbandry

The subjects were three adult brown bears: one male aged 21, and two females both aged 18. They were housed at the Fasano Zoo Safari Park (Apulia, Italy) in a ∼220m^2^ old-style pit surrounded by walls with the ground divided in two portions, one covered by concrete and one made of dirt with some vegetation. The area also included a climbing rock (in the middle) and a small pool. The indoor enclosure consisted of two ∼8m^2^ adjacent and connected rooms. Bears had free access to the indoor and outdoor enclosures 24 hr a day across all seasons, and were locked indoor for a few minutes only during the daily morning cleaning routine. Diet included seasonal fruit and vegetables, fish, and meat, and amount and calories provided throughout the year were not scheduled to vary seasonally, if not in response to a marked winter increase in leftovers (i.e., bears’ decrease in feeding activity was spontaneous and not induced). Drinking water was available at libitum. Feeding schedule would vary depending on the opening schedule of the zoo. In the high touristic season (from April to October), bears were fed several times during the day since part of the feedings would serve as educational shows for the visitors. Differently, during zoo closing months (from November to March) bears were fed once or twice a day. The old-style pit did not prevent the visitors to feed the animals (usually peanuts), despite the presence of prohibition signs and the official staff supervision.

This study was carried out by conducting non-invasive behavioral observations and non-invasive fecal sample collection during the animal daily routine management, and contact or anesthesia was never required. This research was conducted in strict accordance with the recommendations in the "Guidelines for the ethical treatment of nonhuman animals in behavioral research and teaching" (2023, by the ASAB Ethical Committee/ABS Animal Care Committee).

### Behavioral and environmental data collection

Based on previous studies [31, 33, 57–61] an ethogram adapted to captive conditions was developed (52 behavioral patterns). For this study we evaluated a subset of 17 behavioral indicators selected to characterize seasonal phases typical of the hibernation response (such as levels of inactivity/activity and feeding behaviors) and to infer the bear motivational state (such as appetitive feeding and denning behaviors) (Table 1). To ease the comparison of our data to published papers available, we excluded from activity levels all feeding activities. In fact, captive conditions did not allow any bear actual movement related to food search and typical foraging behavior, but just the actual food intake. As a consequence, we provided results for both activity and inactivity levels since together they do not represent 100% of observation time.

**Table 1:** Selected ethogram of brown bear behaviors and behavioral classes.

Behavioral Classes and Behaviors	Definition	References
**INACTIVITY**		
Lying Down	The bear stays with its full weight distributed with haunches on a surface and front arms extended, at most, to the elbows	Andrew et al., 2014
Sitting	The bear stays with back half of weight distributed on ground with front legs extended and paws on the ground	Andrew et al., 2014
Rest with body contact	The bear rest with body contact with another bear	Modified from Montaudouin & Le Pape, 2004
**ACTIVITY**		
Locomotion	The bear walks, without sniffing the ground, runs or climbs	Montaudouin & Le Pape, 2004
Swimming	The bear engages in aquatic activity, moving in a pool, where simple walking would not suffice	Wagman et al., 2018
Standing	The bear stands with three or four paws on the ground and no locomotion	Andrew et al., 2014
Float in water	The bear sits or stand in the pool	Present study
Manipulating object	The bear makes contact with a non-edible object, with any part of the body manipulating its position	Fernandez et al., 2020
Solitary Play	The bear raises or snaps branches, paddles in the water, plays with his own paws. He rolls or runs zigzagging	Montaudouin & Le Pape, 2004
Sniff	The bear sniffs the ground while walking	Modified from Montaudouin & Le Pape, 2004
Affiliative behaviors	The bear is the instigator of, recipient of, or is mutually engaged in active behavior with a conspecific, such as play, grooming and mating	Wagman et al., 2018
Agonistic behaviors	The bear is the instigator of, recipient of, or is mutually engaged in active behavior with a conspecific, such as paw swipes and bared teeth with audible vocalizations, apparently fighting, or chasing	Wagman et al., 2018
Attentive to outside	The bear observes or listen outside the enclosure, watches the visitors, the keeper or the observer with its head and body oriented towards them, or begs for food (i.e., appetitive feeding behavior)	Modified from Montaudouin & Le Pape, 2004
Appetitive feeding behavior	The bear begs for food while is sitting or standing (sometimes waving its front paws) in the proximity zone to visitors, staring at them (more than 5 s) as they walk past the exhibit. Begging can also be directed to the keeper	Modified from Montaudouin & Le Pape, 2004; Podturkin, 2022
Denning behaviors	The bear engages in transporting nest materials, maintening the nest and digging	Modified from Friebe et al. 2001; Wagman et al., 2018; Kim et al., 2020
**FEEDING BEHAVIORS**		
Feed	The bear ingests edible material	Vickery & Mason, 2004
Manipulate food items	The bear engages in any nonstereotyped manipulation (but not actual ingestion) of edible food materials	Vickery & Mason, 2004
**OUT OF SIGHT**		
Out of sight	The bear is not visible (or behavior not discernible) to the observer	Andrew et al., 2014

Observations were conducted by the first two authors between July 2021 and April 2022 with a stop of data collection in December, January and March. A pilot study of 2 weeks preceded the observations, after which inter-observer reliability was reached (Cohen’s K over 90%). Direct observations were carried out during zoo opening hours (9 am ‐ 6 pm), with 4 to 5 observation hours per day, 4 to 5 days a week. We used a 15-minute focal animal sampling (4 to 5 repetition per day per individual) and used a combination of one-zero and instantaneous recording techniques [62] (15 second intervals). We excluded from the analyses the sessions with more than 10% observation time with bears “out of sight”, finally obtaining a total of N = 1330 focal observations (an average of N = 443/individual) for a total of N = 322,5 hours of observations (an average of N = 110.75/individual). The order the three bears were observed in a given day, was randomized each day and maintained throughout that day. Observational sessions were calendarized in order to obtain an equal number of records per individual as for the day of the week and the time slots.

The number of visitors was recorded both at the beginning and at the end of each focal observation. Daily average temperatures were collected using a USB data logger (EasyLog, EL-USB-2-LCD), while proportion of daylight hours were calculated based on photoperiod data reported for that specific geographic area (https://www.calendariando.it/alba-e-tramonto/fasano).

### Fecal sample collection and fecal cortisol metabolites (FCM) quantification

Fecal samples were collected in the morning during the daily cleaning routine. Scat freshness was assessed based on consistency and appearance, and for identification of individual feces each subject was fed twice a day with food filled with differently colored corn [63]. Immediately after collection, samples were stored at -20˚C until laboratory analysis, after labelling with date/hour of collection and individuals’ names. A set of 142 fecal samples (50, 47, 45 for the male and the two females, respectively) was sent to the Veterinary Department (Torino University, Italy) for FCM analysis. Ethanol extraction and determination of corticosteroids in the feces were carried out as previously reported [64, 65] using a multispecies cortisol enzyme immunoassay kit (K003; Arbor Assays, Ann Arbor, MI) validated for dried fecal extracts. All analyses were repeated twice. Cortisol kit cross reactivity, according to the manufacturer, was: 100% with cortisol, 18.8% with dexamethasone, 7.8% with prednisolone, 1.2% with corticosterone, and 1.2% with cortisone, consequently we referred to hormonal results as fecal cortisol metabolites (FCM). The inter- and intra-assay coefficients of variation were less than 10% (6% and 8% respectively). The test’s sensitivity was determined by measuring the least amount of hormone standard consistently distinguishable from the concentration of standard zero and was calculated to be 17.3 pg/mL. Serial dilutions (1:4, 1:8, 1:16, and 1:32) of fecal samples were assayed to test for parallelism against the standard curve (r^2^ = 0.985). The mean recovery rate of cortisol added to dried feces was 94,8% (n = 6). FCM concentrations are expressed as ng/g of dry feces.

### Body condition score (BCS)

BCS data were collected from June 2021 to April 2022 using a noninvasive photograph-based method developed and validated for wild brown bears [66]. In order to extract morphometric measurements (in pixels) an average of 4 photos were collected twice a month (about every two weeks) for each bear and each photo was measured 3 times (blind measurements, ImageJ software, version 1.537t [67]) to finally obtain a mean score per photo. The BCS was given by the torso height:horizontal torso length ratio (TH:HTL). Only lateral, non-tilted photographs were used (N = 135) and all pics not matching the measurability requirements of the method were excluded.

### Statistical analyses and four phases determination

Our behavioral dataset was represented by focal animal observations combined to obtain daily frequencies. Hormonal dataset was represented by about 3 samples per animal per week, and BCS included all data sampled (i.e., measurements deriving from four selected photo per animal every two weeks). We investigated the variation of behavioral, hormonal, and morphological data by dividing our study period into phases that would try to mimic those seasonal phases faced by bears in the wild, specifically characterizing their activity patterns as related to the hibernating adaptation. We therefore combined literature data with information deriving from a preliminary exploration of our data, focusing on bear inactivity levels (our best and potential indicator of an approaching and/or full experience of hibernation) and checking for a seasonal trend. Based on this we identified the maximum level of inactivity (∼76%) in February, and this month was potentially labeled as “hibernation phase”. Based on the following decrease of inactivity (i.e., the bears became more active) observed in April, we labeled this month as “hypophagic phase”. To identify phases potentially faced by bears during months preceding the hibernation phase, we had to deal with a smooth and progressive bear inactivity increase, whose beginning was therefore impossible to identify. By observing monthly plots of activity and inactivity levels, we arbitrarily identified the separation between the hyperphagic and the transition phases when the inactivity and activity levels (i.e., monthly medians) switched, going from activity being higher than inactivity, to the opposite, having inactivity exceeding activity (between September and October). Based on this, preceding summer months from July to September, were labeled as the “hyperphagic phase”, followed by October and November which were labeled as the “fall transition phase”. To ease the presentation of the results, now on phases will be cited as hyperphagia, transition, hibernation and hypophagia, which were then used as time periods to analyze our response variables and environmental covariates.

Analyses were completed in R Version 4.2.1 [68]. Since none of our data sets met the normal distribution assumptions (Shapiro-Wilk tests), we performed a data transformation that, however, only worked for FCM whose log transformed values successfully approximated normality. We tested for variation in visitor numbers across months by using Kruskal–Wallis analysis of variance (ANOVA) on rank tests and post-hoc pairwise comparisons (using Dunn’s Method) were implemented. To test the potential influence of environmental variables, reported in the wild as likely triggers of the bear typical activity/inactivity patterns, we checked for interaction with temperature and photoperiod by running regression using the package ‘mgcv’.

Generalized linear mixed models (GLMM) were applied to examine if our response variables (i.e., activity, inactivity, FCM, and BCS) would vary throughout the four phases, months, and half-months (depending on the variable). As for behaviors and BCS we run GLMMs using the package ‘glmmTMB’ [69] using the beta distribution, which is appropriate for proportion data [70]. In order to use the beta distribution in R, and limited to the behavioral dataset, we converted all zeros and all ones in the dataset to 0.0000001 and to 0.9999999 respectively (e.g., see [54]). As for FCM we run GLMMs using the package ‘lme4’ [71] using the normal distribution. Models are described in details hereafter. We ran two GLMMs for each one of the response variables. For activity and inactivity, analyzed separately, one model included four phases and the other one included months as fixed factors. When considering FCM we included phases and half-months as fixed factors, while for BCS we included four phases and half-months as fixed factors. Bear identity was included as random factor in each model tested. A Tukey post hoc test (function ‘glht’ in ‘multcomp’ package) was used for all analyses.

Based on a multifactorial environmental scenario possibly affecting bear behaviors, we built additional statistical models considering environmental covariates. For both activity and inactivity levels, in addition to the four phases (our grouping variable), each environmental covariate (i.e., photoperiod, temperature, visitor numbers) was included singularly and in all possible combinations, resulting in 7 models of increasing complexity. Phases, temperature, photoperiod and visitor numbers were used as fixed factors and bear identity as a random factor. Interactions among fixed factors were also included in model building. Final models were obtained by removing non-significant factors and interactions and later compared using AIC scores (function ‘aictab’ in ‘AICcmodavg’ package) to determine the best fitting model. Before running GLMMs we checked for multicollinearity with the variance inflation factors [72]. For FCM levels, we build one additional model including phases and visitor numbers (as a covariate possibly affecting stress levels) as fixed factor and bear identity as a random factor.

## Results

### Behavioral trends

**Fig 1:**
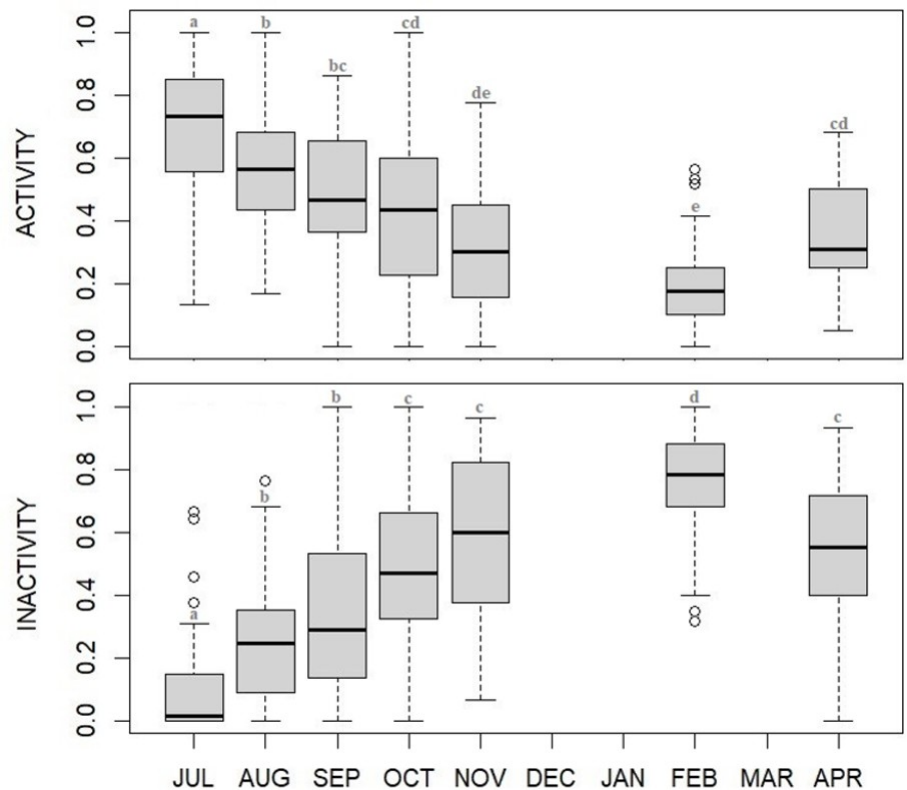
Monthly changes (median plot) in the proportion of time spent in activity and inactivity by captive brown bears during the study period (July 2021-April 2022). High summer levels of bear activity gradually decrease toward winter months, whereas inactivity levels gradually increase during fall and wintertime and overcoming activity levels in October, at the beginning of potential fall transition phase. Statistical details reported in S1 and S2 Tables.

**Fig 2:**
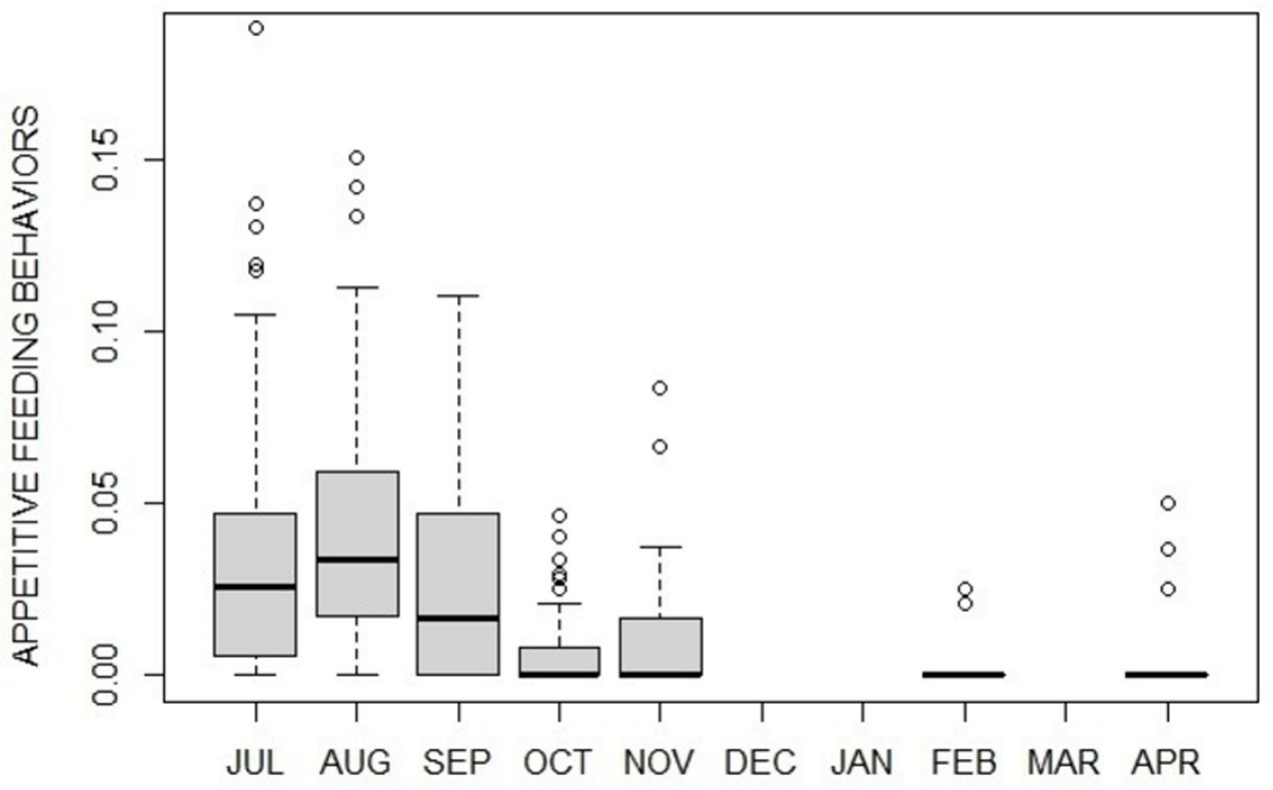
Monthly changes (median plot) in the proportion of time spent in appetitive feeding behaviors levels by captive brown bears. Appetitive behavior, as proxy of motivation to feeding, is high in summer months (i.e., potential hyperphagia), and low in fall, winter and spring (i.e., potential fall transition, hibernation and hypophagia, respectively).

**Fig 3:**
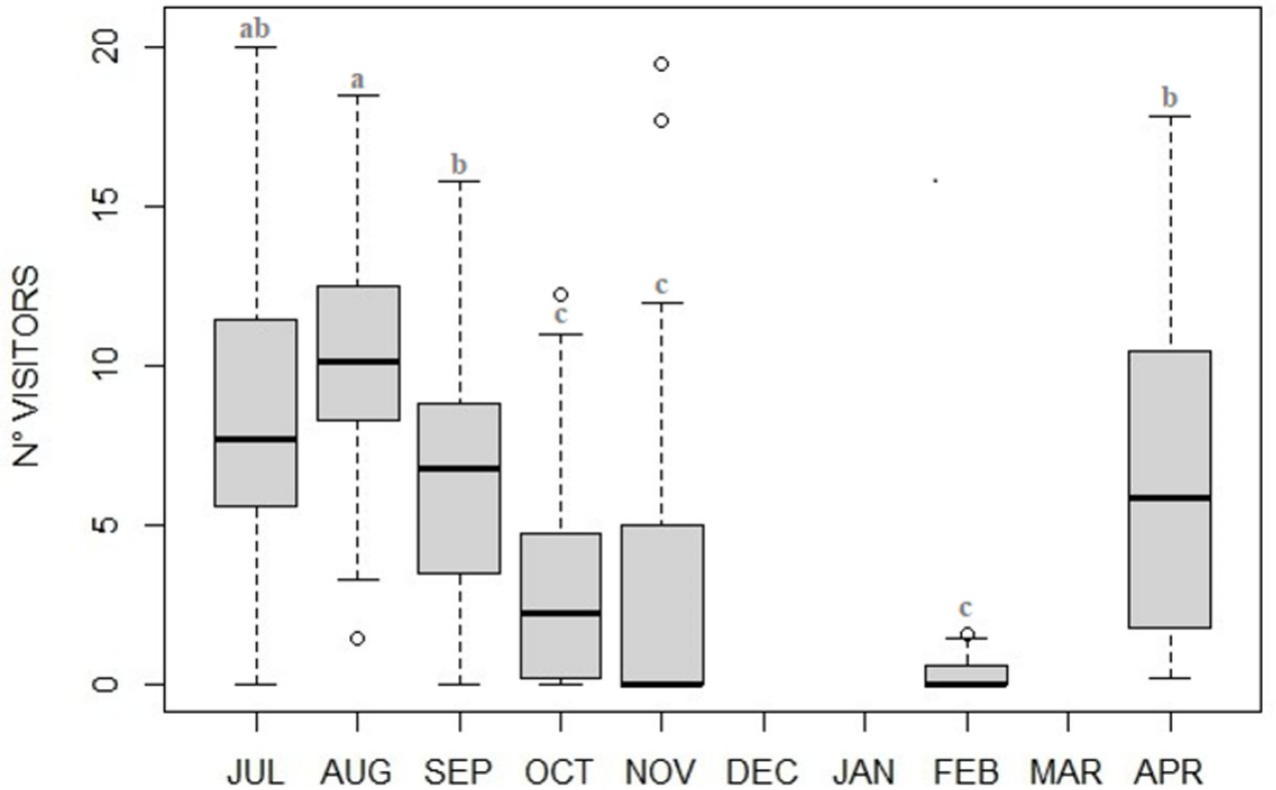
Monthly changes (median plot) of visitor numbers at the bear exhibit during observation sessions. Visitor numbers is significantly different throughout the months, showing highest turnover in summertime (i.e., potential hyperphagia) and a significant decrease in fall (October-November, i.e., potential fall transition). Statistical details reported in S3 Table.

Overall levels of activity and inactivity varied between months (activity: F_6_ = 26.78, p<0.001; inactivity: F_6_ = 48.40, p<0,001), with activity being higher during summer and then gradually decreasing toward hibernation, whereas inactivity gradually increasing and peaking in February (Fig 1A and 1B and S1 and S2 Tables). Appetitive feeding behavior presented higher values during summer months (hyperphagia) with a sharp decrease in the following fall, winter and spring months (Fig 2). As visitor numbers resulted significantly different between months (Fig 3, χ^2^_6_ = 153.23, p<0.001; S3 Table) with a drastic fall in October we tested for a possible correlation between visitor numbers and appetitive feeding behavior frequency, resulting in a highly significant positive correlation (ρ = 0.61, p<0.001). Feeding behaviors varied between months (F_6_ = 9.29, p<0.001; S4 Table) being higher during summer compared to the other months.

**Fig 4:**
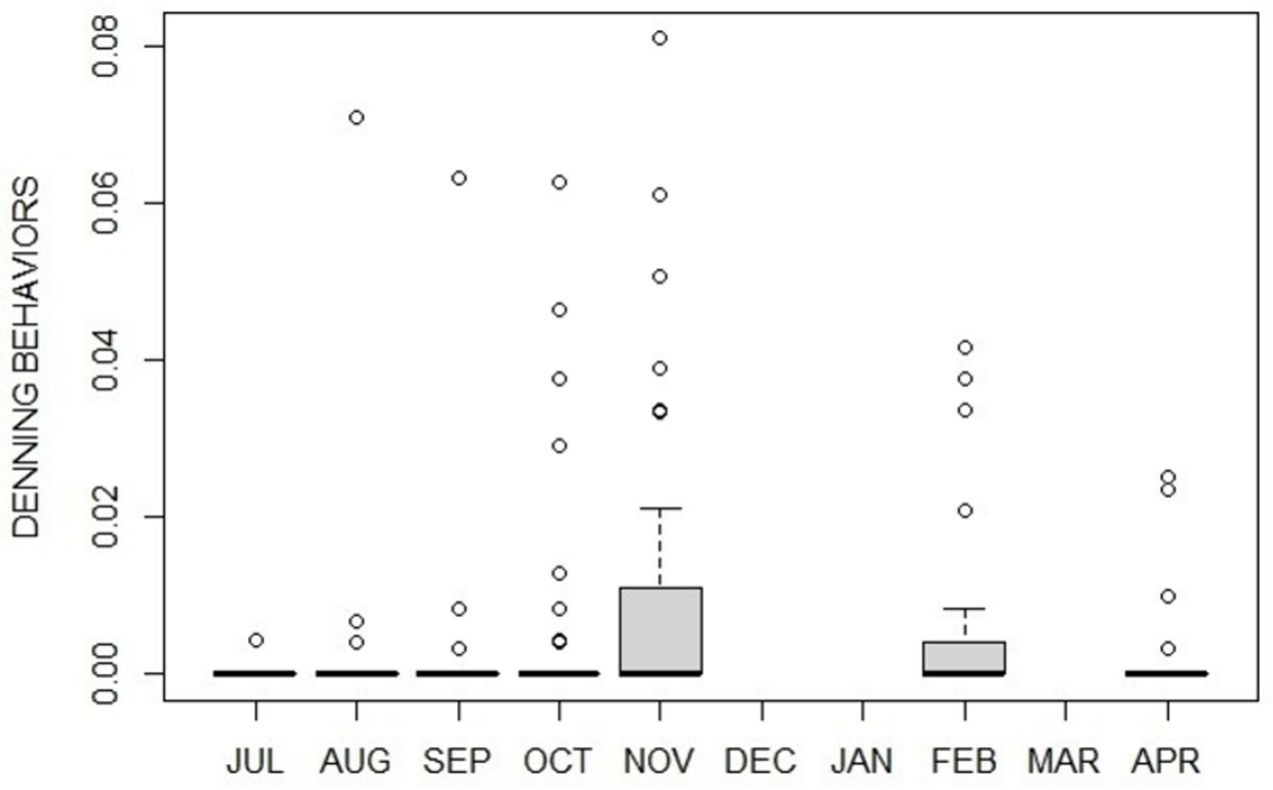
Monthly proportion (median plot) of denning behaviors by captive brown bears. Denning behaviors are extremely rare, however they mostly occur during potential fall transition.

Denning behaviors mainly occurred during fall transition (October-November) and hibernation (February) (Fig 4).

Temperature and photoperiod were positively correlated (as expected, ρ = 0.73, p<0.0001). Regression models showed that activity was positively associated with both temperature and photoperiod, with about a 30% of the variance explained (R^2^ = 0.27, p<0.001, deviance explained = 27.4% and R^2^ = 0.31, p<0.001, deviance explained = 32.5% respectively) in opposition to inactivity levels, negatively associated with both temperature and photoperiod, however with better results, having about a 40% of the variance explained (R^2^ = 0.39, p<0.001, deviance explained = 39.9% and R^2^ = 0.41, p<0.001, deviance explained = 43.2% respectively). Visitor numbers also positively correlated to temperature (R^2^ = 0.32, p<0.001, deviance explained = 34.3%) and photoperiod (R^2^ = 0.39, p<0.001, deviance explained = 40.4%) therefore this variable was included.

**Fig 5:**
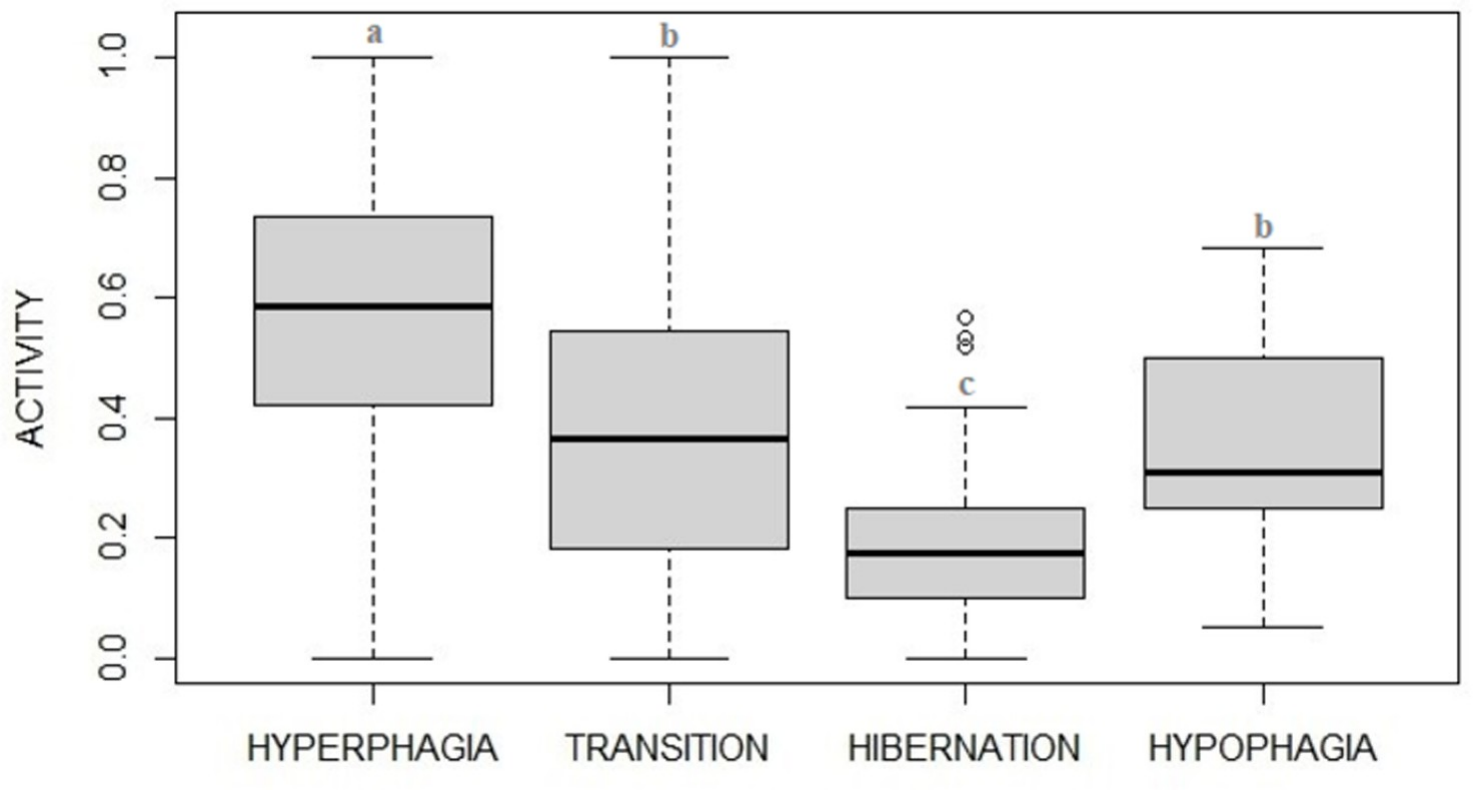
Proportion (median plot) of time spent in activity by captive brown bears in the four phases. Activity levels appears to fit with potential hibernation cycle described in brown bears, with lowest levels shown in winter (i.e., potential hibernation phase). Statistical details shown in Table 2.

Levels of activity varied between phases (Fig 5, F_3_ = 38.24, p<0.001) being higher during hyperphagia, decreasing during fall transition, reaching a minimum during hibernation and then increasing back in hypophagia (Table 2). Among the candidate models, two high ranked models (within delta AIC≤2) were found both suggesting that activity was best predicted by photoperiod and to a lesser extent by the interaction between visitors and phases, with the highest ranking including phases, photoperiod, and visitors as fixed factors (AIC = -121.21 and a weight of 0.72) and the second ranking including phases, photoperiod, temperature and visitors (AIC = -119.22 and a weight of 0.26) (Table 3).

**Table 2:** Post hoc Tukey’s test results for activity between phases.

Phases	Estimate ± S.E.	z value	P
Transition ‐ Hyperphagia	-0.19071 ± 0.02720	-7.012	<0.001
Hibernation ‐ Hyperphagia	-0.37839 ± 0.04200	-9.008	<0.001
Hypophagia ‐ Hyperphagia	-0.21039 ± 0.04200	-5.009	<0.001
Hibernation ‐ Transition	-0.18768 ± 0.04456	-4.212	<0.001
Hypophagia ‐ Transition	-0.01968 ± 0.04456	-0.442	0.970
Hypophagia ‐ Hibernation	0.16800 ± 0.05486	3.062	0.011

**Table 3:** GLMM results explaining the variation in bear activity throughout the four phases, also considering variations in environmental variables such as photoperiod, temperature and visitor numbers.

Models	Explanatory variables	DF	F	P	^a^K	^b^AIC	^C^Delta AIC	^d^Weight	^E^Cum. Wt	^f^LL
**ACTIVITY**										
∼ Phases + Ph + Vis + Phases*Vis	Phases	3	2.25	0.08283	11	-121.21	0.00	0.72	0.72	71.61
	Photoperiod	1	39.78	9.52e-10						
	Visitors	1	0.73	0.39415						
	Phases*Visitors	3	3.63	0.01324						
∼ Phases + Ph + T + Vis + Phases*Vis	Phases	3	1.64	0.18102	12	-119.22	2.00	0.26	0.98	71.61
	Photoperiod	1	27.39	3.03e-07						
	Temperature	1	0.001	0.97492						
	Visitors	1	0.71	0.39721						
	Phases*Visitors	3	3.61	0.01363						
∼ Phases + Ph	Phases	3	12.28	1.25e-07	7	-113.49	7.72	0.02	0.99	63.75
	Photoperiod	1	45.29	7.82e-11						
∼ Phases + Ph + T	Phases	3	7.09	0.0001248	8	-111.54	9.67	0.01	1.00	63.77
	Photoperiod	1	0.05	0.8219964						
	Temperature	1	32.73	2.42e-08						
∼ Phases + T + Vis + Phases*Vis	Phases	3	1.82	0.141881	11	-94.17	27.04	0.00	1.00	58.09
	Temperature	1	11.33	0.000858						
	Visitors	1	0.69	0.404448						
	Phases*Visitors	3	3.89	0.009420						
∼ Phases + Vis + Phases*Vis	Phases	3	11.09	6.01e-07	10	-84.76	36.46	0.00	1.00	52.38
	Visitors	1	1.23	0.26839						
	Phases*Visitors	3	3.45	0.01698						
∼ Phases + T	Phases	3	1.45	0.2285887	7	-81.89	39.33	0.00	1.00	47.94
	Temperature	1	11.34	0.0008478						

Predictors’ abbreviations: Ph, photoperiod; T, temperature; Vis, visitors.

Model characteristics:

^a^K, number of variables included

^b^AIC, Akaike’s information criterion

^c^Delta AIC, difference in AIC between the model with the lowest AIC and the target model

^d^Weight, model probabilities

^e^Cum.Wt, cumulative weight; LL, log-likelihood of each model.

**Fig 6:**
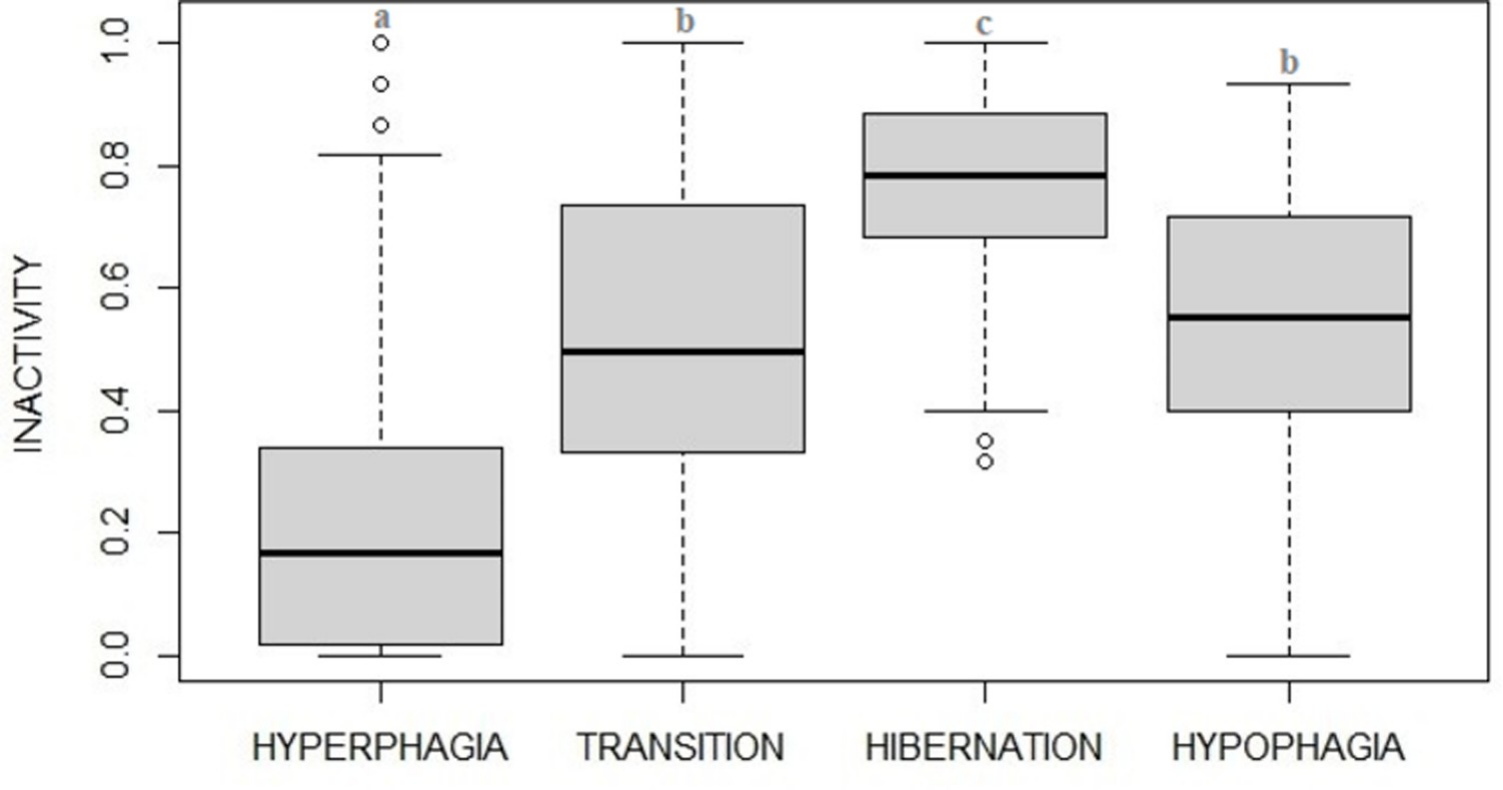
Proportion (median plot) of time spent in inactivity by captive brown bears in the four phases. Inactivity levels appear to fit with potential hibernation cycle described in brown bears, with highest levels shown in winter (i.e., potential hibernation phase). Statistical details shown in Table 4.

Levels of inactivity also varied between phases (Fig 6, F_3_ = 70.33, p<0.001) with levels being at the lowest during hyperphagia, increasing during fall transition, reaching the maximum during hibernation and then decreasing in hypophagia, as expected (Table 4). Among the candidate models, analysis found two high ranked models (within delta AIC≤2), the best one explaining the variation in inactivity (AIC = -91.03 and a weight of 0.73) suggested that inactivity was best predicted by phases, photoperiod and visitor numbers (photoperiod: F_1_ = 45.446, p<0.001; visitors: F_1_ = 20.357, p<0.001; Table 5), while the second one (AIC = -89.04) also included temperature among fixed factors (Table 5).

**Table 4:** Post hoc Tukey’s test results for changes in inactivity proportion between phases.

Phases	Estimate ± S.E.	z value	P
Transition ‐ Hyperphagia	0.29130 ± 0.02926	9.954	< 0.001
Hibernation ‐ Hyperphagia	0.53389 ± 0.04519	11.813	< 0.001
Hypophagia ‐ Hyperphagia	0.31911 ± 0.04519	7.061	< 0.001
Hibernation ‐ Transition	0.24259 ± 0.04794	5.060	< 0.001
Hypophagia ‐ Transition	0.02781 ± 0.04794	0.580	0.93489
Hypophagia ‐ Hibernation	-0.21478 ± 0.05903	-3.639	0.00132

**Table 5:** GLMM results explaining the variation in bear inactivity throughout the four phases, also considering variations in environmental variables such as photoperiod, temperature and visitor numbers.

Models	Explanatory variables	DF	F	p	^a^K	^b^AIC	^C^Delta AIC	^d^Weight	^e^Cum.Wt	^f^LL
**INACTIVITY**										
∼ Phases + Ph + Vis	Phases	3	19.85	7.98e-12	8	-91.03	0.00	0.73	0.73	53.51
	Photoperiod	1	45.45	7.32e-11						
	Visitors	1	20.36	9.03e-06						
∼ Phases + Ph + T + Vis	Phases	3	9.51	4.94e-06	9	-89.04	1.99	0.27	1.00	53.52
	Photoperiod	1	30.96	5.59e-08						
	Temperature	1	0.001	0.9165						
	Visitors	1	20.31	9.27e-06						
∼ Phases + Ph	Phases	3	20.96	1.19e-12	7	-72.89	18.14	0.00	1.00	43.45
	Photoperiod	1	55.16	1.01e-12						
∼ Phases + Ph + T	Phases	3	9.12	8.29e-06	8	-70.89	20.14	0.00	1.00	43.45
	Photoperiod	1	0.0001	0.9978						
	Temperature	1	38.17	1.97e-09						
∼ Phases + T + Vis	Phases	3	1.79	0.1478700	8	-60.96	30.07	0.00	1.00	38.48
	Temperature	1	13.12	0.0003381						
	Visitors	1	27.27	3.19e-07						
∼ Phases + Vis	Phases	3	30.16	<2.2e-16	7	-49.90	41.13	0.00	1.00	31.95
	Visitors	1	29.34	1.19e-07						
∼ Phases + T	Phases	3	3.24	0.0224186	7	-36.27	54.76	0.00	1.00	25.14
	Temperature	1	15.07	0.0001255						

Predictors’ abbreviations: Ph, photoperiod; T, temperature; Vis, visitors.

Model characteristics:

^a^K, number of variables included

^b^AIC, Akaike’s information criterion

^c^Delta AIC, difference in AIC between the model with the lowest AIC and the target model

^d^Weight, model probabilities

^e^Cum.Wt, cumulative weight

^f^LL, log-likelihood of each model

Also, feeding behaviors varied between phases (F_3_ = 14.76, p<0.001; S5 Table) with levels being higher during hyperphagia compared to the other phases.

### Fecal cortisol metabolites

**Fig 7:**
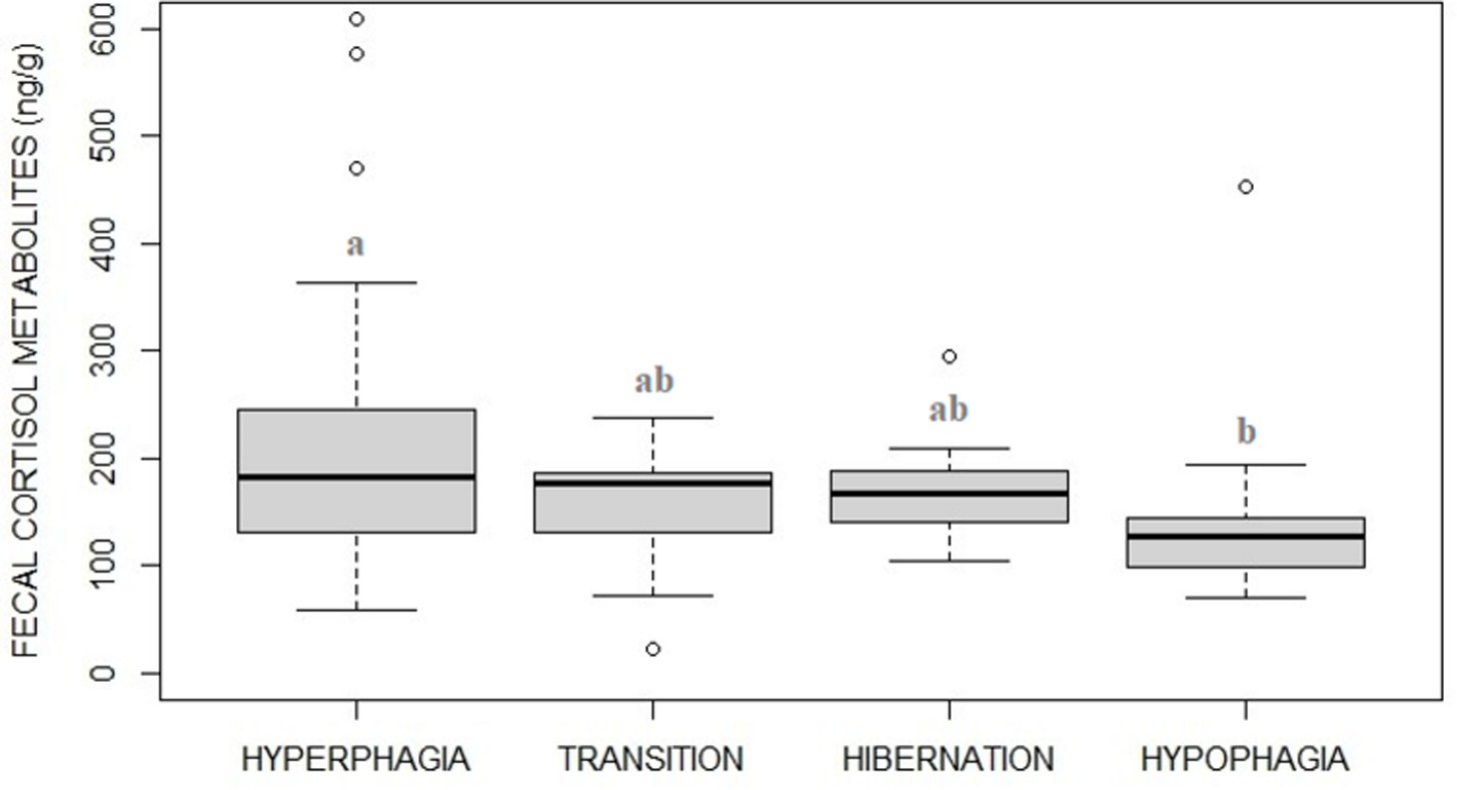
Fecal cortisol metabolite levels (FCM) of captive brown bears in the four phases. FCM levels were higher in hyperphagia compared to hypophagia.

FCM levels varied between the four phases (Fig 7, F_3_ = 4.63, p = 0.0039) with the post-hoc revealing FCM concentration being higher during hyperphagia compared to hypophagia (p = 0.0049). When including visitor numbers in the model, FCM levels were significantly predicted by phases only (phases: F_3_ = 4.59, p = 0.0045; visitors: F_1_ = 0.46, p = 0.495).

### Body condition score

**Fig 8:**
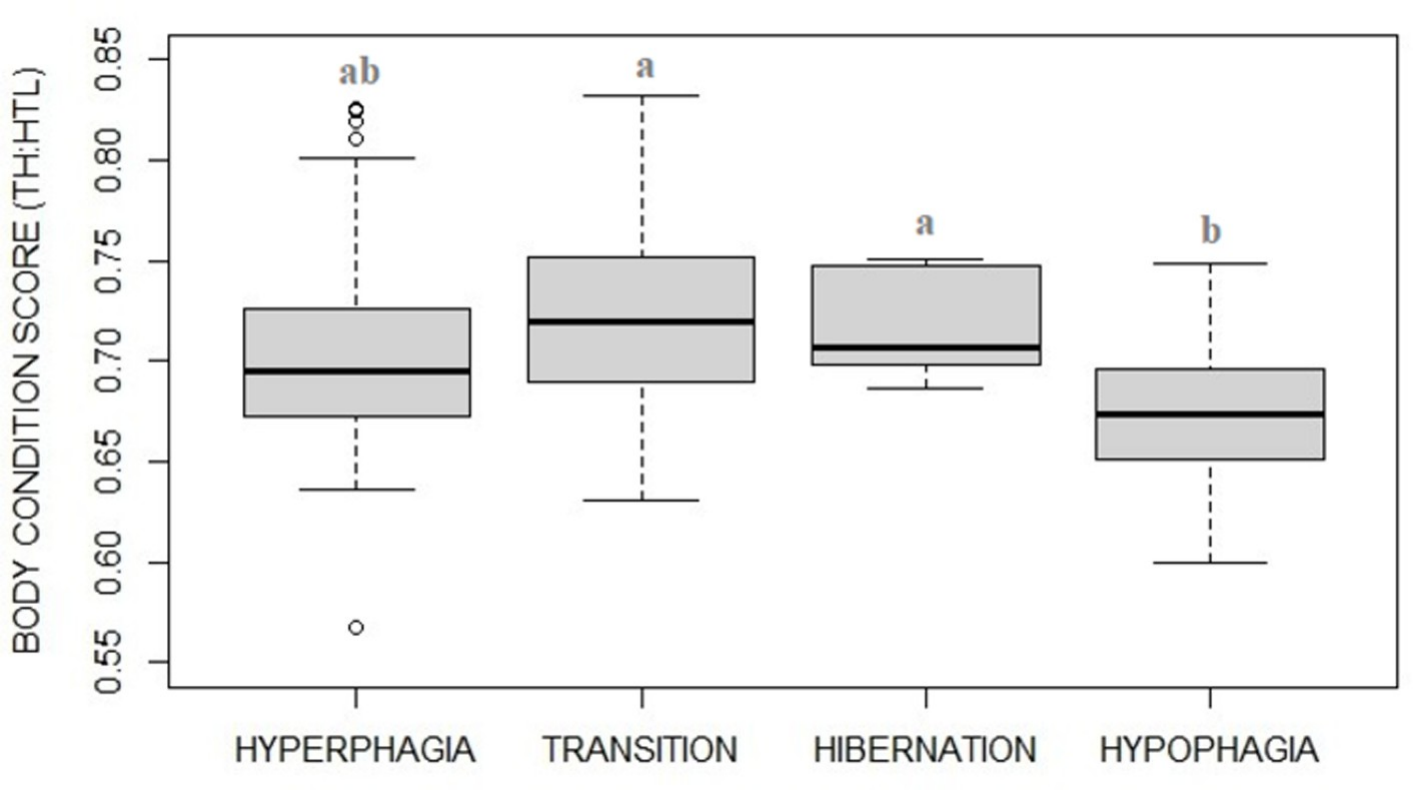
Body condition score (BCS, median plot) of captive brown bears in the four phases. BCS varied throughout the phases being higher during fall transition and hibernation compared to hypophagia, reflecting a loss of body mass during winter. BCS was calculated by torso height:length ratio (TH:HTL) from photographs taken in the outdoor area.

**Fig 9:**
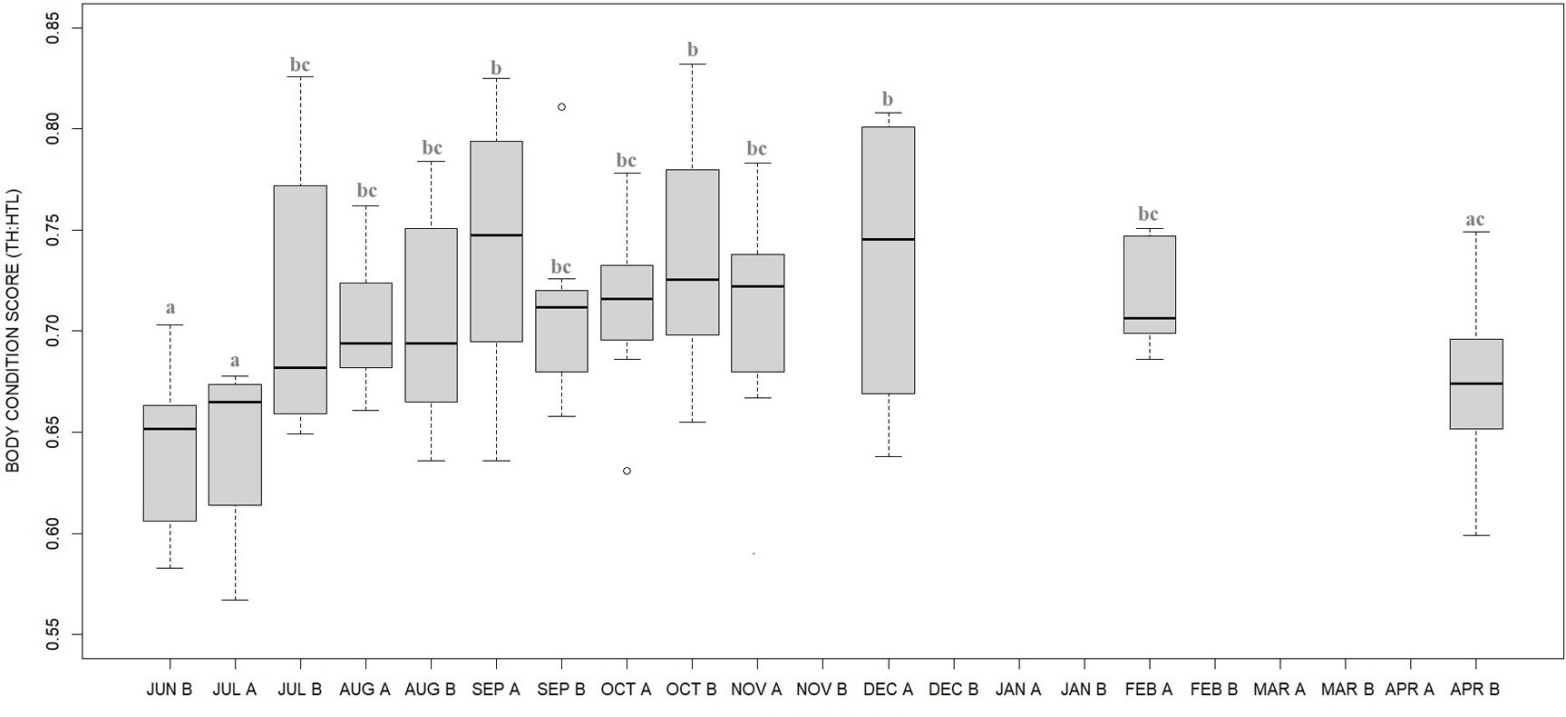
Semi-monthly changes in body condition score (BCS, median plot) of captive brown bears. BCS increased from early (June-July) to mid-late summer (August-September, reflecting a weight gain during the hyperphagic period. The BCS was calculated by torso height:length ratio (TH:HTL) from photographs taken in the outdoor area. Letters A and B refer to the half-month periods as photographs were collected twice a month (about every two weeks).

BCS varied between the four phases (Fig 8, F_3_ = 5.4, p = 0.0015) with body condition scores being higher during fall transition (p = 0.0153) and hibernation (p<0.001) compared to hypophagia, reflecting a loss of body mass during winter. However, when analyzing BCS more in details between half-months (Fig 9, F_12_ = 7.48, p<0.001; S6 Table), there was an increasing from early (June, July) to mid-late summer (August, September) reflecting a body mass gain during the hyperphagic period. BCS showed a 11% increase from spring (June) to autumn (November) and a 6% decrease from autumn to spring (April).

## Discussion

Despite the year-round unvaried management conditions, captive brown bears in this study did show seasonal behavioral, hormonal, and morphological patterns which can be assimilated to the bear natural predisposition to hibernate. The integrated approach combining different categories of animal-based measures (behaviors, hormones, and body condition) and environmental covariates was effective in providing an overall insight into the changes occurring in captive brown bears over the seasons.

Both activity and inactivity patterns showed a clear trend, either when observed divided in phases (hyperphagia, transition, hibernation, hypophagia) or more in details when grouped by months. Highest levels of activity were recorded in the hyperphagic phase, whereas highest levels of inactivity were recorded in the hibernation phase. The gradual decrease in the frequency of active behaviors and gradual increase of inactivity from summer to winter observed in a middle (temperate) latitude zoo, is in line with the ecology of the brown bears (e.g., [3]) whose annual cycle is strongly driven by seasonality (e.g., [17]).

Seasonality, as described by temperature and photoperiod, has been reported in the literature as affecting bear activity/inactivity patterns [3, 4, 12]. In fact, low ambient temperatures may affect timing of den entry, as driver of reduction in body temperature therefore determining an earlier den entry in colder years [4]. Conflicting results are reported for the role of photoperiod in wild brown bears, with its key effects (together with snow depth) reported in Evans and colleagues [4], while dismissed by [3] (though measured as weekly daylight average). In order to explore in detail our data, and for better comparison to other studies, we analyzed both environmental variables independently, though strongly correlated. In fact, regression showed the activity pattern positively related and inactivity pattern negatively related to both temperature and photoperiod.

In this study, between September and October a switch between activity and inactivity levels was observed (with inactivity exceeding activity levels). We also observed first appearance of distinctive denning behaviors (i.e., transport nest materials, digging and nest maintenance) which naturally represent the bears’ need to set up a nest in preparation for winter den and, as such, they are considered characteristic of the fall transition [14]. In zoos where hibernation is induced, nest materials are provided during fall [12, 33], whereas, in this study, the bears spontaneously uprooted plants naturally growing in the outdoor enclosure, confirming our initial identification of these months as the transition phase. Previous months (from July to September) were validated as hyperphagic phase by both the levels of feeding behaviors, significantly higher during hyperphagia than in any other phase/month, and the trend of the appetitive feeding behaviors, high until September then falling in October. Following transition, and during hibernation, wild bears exhibit continuous dormancy for months without eating, drinking, defecating, or urinating [16]. In our study, although the animals would show some levels of activity with very exiguous feeding behaviors, February was the month characterized by the highest peak of inactivity as also described by literature, which definitely reports February as part of the hibernation phase both in North America and Eurasia, including in areas located at our latitude [18]. Unfortunately, we do not know whether bears actually spent a period of complete inactivity (i.e., without eating, drinking, defecating, or urinating) in winter months of December/January, due to a lack of data, however we know they were sleeping most of the time (keepers’ pers. comm.). Finally April, our tentative hypophagic phase, was characterized by an activity resumption after winter months with feeding behaviors still significantly lower compared to summer, as expected in a typical hypophagic phase [17].

In this study, we aimed at clarifying whether a geographically temperate captive setting with access to constant food resources and no kind of hibernation management can, and if yes, how much, affect the natural hibernation response in brown bears, and, in turn, if and how this should be considered in relation to welfare. To do this we opted for an integrative approach based on: animal indicators whose seasonal pattern related to hibernation is known (behavior, glucocorticoids, and BCS; e.g., [3, 7, 38]), environmental covariates known to affect beginning and duration of denning and hibernating responses (e.g., climatic ones such as temperature and photoperiod), and, finally also visitor numbers, as inevitable factor affecting captive animal behavior.

The best and simplest model explaining the patterns of activity/inactivity in the four phases, identified photoperiod and visitors as crucial variables, while the temperature proved influential only in simpler models, when it was considered either as the only covariate or coupled with another covariate only (either photoperiod or visitors). Besides the strong correlation between temperature and photoperiod at a temperate latitude, we hypothesized that the high daily variability of temperature likely affected behavior more on a day-to-day basis, while the whole pattern of steady decrease/increase in activity/inactivity levels throughout the months was better described by a less fluctuating variable, with a more linear and constant progression, as the photoperiod (S1 Fig, for temperature and photoperiod variations during the study). The influence of both the visitor numbers and photoperiod on the bears’ activity may require some explanation. In zoos where animals are only visible to the public in the outdoor enclosure (as in Italy), the “zoo seasons” are described by a combination of environmental variables (visitors, temperature, photoperiod) which altogether strongly covary, either positively, with plenty of visitors, high temperatures and long daylength (during spring and summer, the zoo opening seasons) or negatively, with no/few visitors, low temperatures, short daylength (during fall and winter, corresponding to zoo opening only on holidays and weekends). This entangled context might have been contributing especially to both hyperphagia and fall transition behavioral response. When compared to the hyperphagic phase observed in wild Italian brown bears (late summer ‐ early fall, *U*. *a*. *marsicanus* [13]) that of our subjects resulted slightly anticipated in mid- late-summer (July, August, September) just when there was a peak in turnout of visitors (i.e., high season at the zoo). It is likely that the increasing food intake (as estimated by the increased time spent feeding) right in these summer months, depended on the large food amount available due to that provided by the visitors who, although forbidden, kept feeding animals (also favored by the old-pit style enclosure, typically missing barriers). Hyperphagia, in addition, is also characterized by a change in the nutritional composition of the diet [13] and wild bears, in order to gain fat, mainly rely on high calorie food. Accidentally, peanuts were the most common food provided by visitors, and their high caloric, high nutritional value food characteristics [73] might have partially met the bears’ extra calorie and fat requirements, as typical in the wild around this time of the year. Bear higher feeding motivation was also supported by food related appetitive behavior which was higher during summertime.

Extra food availability might have also conditioned bear activity levels, that showed high despite temperature exceeding 30°C. In fact, although wild bears are reported avoiding hottest times of day by typically reducing midday activity (from 20°C onwards in grizzly bears [74], from 23°C onwards in American black bears [75]) they may also remain active at high temperatures (up to 40.1°C), specifically when high energy resources are at stake (e.g., berries, reported in grizzly bears [3]). By manipulating pattern and schedule of the light/dark and food availability cues respectively in captive bears, Ware and colleagues [12] demonstrated a bear seasonal sensitivity to both cues, with their relative effects on activity patterns being time of the year dependent. While photoperiod (i.e., a daylength compression) was powerful to affect activity patterns around hibernation phase, food availability was effective during the active phase, even prompting bears to switch from diurnal to nocturnal activity (i.e., adding nightly feedings) (for food and bear behavioral ecology see [76]). In sum, a temporal reorganization of brown bear activity pattern driven by food availability at both the time-of-day and season levels might well reflect the animals’ behavioral and ecological flexibility to rapidly cope with changing environmental conditions, a conditional plasticity that enables individuals to readily exploit resources when available (e.g., [77, 78]).

Similarly to hyperphagia, this study fall transition (from the beginning of October until mid-November) is slightly anticipated compared to that in wild brown bears (from the end of October throughout November [14, 20]). Fall transition anticipation, however, was likely not only a direct consequence of a shifted hyperphagia, since it also corresponded to an astonishing mix accidentally appropriate for the shift from hyperphagic to fall transition phase: a drastic visitor numbers’ drop, a concurrently extra food provided drop, and a shortening of daylength. Our best model supported the role of both visitor numbers and photoperiod, however, at this time, we are unable to decouple the seasonal effect of visitors (and the entangled extra food provided), from the seasonal effects of the other independent environmental variables, since we cannot take advantage of a control condition to make a comparison (see for example [54]).

In our study subjects, we expected that the abundant, non-seasonal and year-round available domestic food resources resulted in flattening potential differences in both FCM and BCS measurements between phases. By comparing two wild brown bear populations, higher levels of FCM in hyperphagia as compared to hypophagia were only found in the population feeding on seasonally fluctuating wild food, and not in that one feeding on easy year-round available human-provided domestic food (i.e., corn and grains targeted at ungulates) [38]. It was hypothesized that the dramatical alteration of the seasonal nutritional intake due to year-round availability of domestic food would impact on wild bear hormonal patterns [38]. Nevertheless, a seasonal BCS fluctuation was found in year-round fed captive black bears, explained by independent seasonal insulin resistance [30]. In our bears, contrary to initial expectations, both FCM and BCS showed a pattern similar to that described in wild naturally feeding and naturally hibernating bear populations [7, 38]. In fact, FCM resulted significantly higher during hyperphagia than hypophagia, and BCS (as a proxy of a gain of fat) resulted higher in hyperphagia and fall transition than in hypophagia (increasing from June to September, remaining steady until December, and later decreasing in April) as expected after supposed hibernation. In the wild, hyperphagic behavior invariably makes brown bear BCS increasing from summer to the fall [7, 66, 79] and seasonality in cortisol levels is functional to the whole hibernation response. During the hyperphagic phase, in fact, bears need to gain fat and an increasing cortisol may support lipogenesis by indirectly and positively affecting appetitive feeding behavior and food intake (via other hormones and neurotransmitters: e.g., neuropeptide Y, proopiomelanocortin, and/or Agouti-related protein [43]). On the contrary, during hibernation the cortisol function turns into the nearly opposite lipolysis. During assisted hibernation, serum cortisol concentration levels in captive grizzly bears was found 366% higher than during the hyperphagic phase levels [39] due to its catabolic role necessary to provide energy during prolonged winter fasting (see also [46]) therefore explaining progressive winter loss of fat. Supposed mechanisms explaining these seasonal, somehow contrasting, cortisol functions, have been hypothesized as either likely depending on its absolute concentration or determined by the influence of other physiological parameters [43].

FCM levels in our study, however, lacked a significant winter increase, and differences between hyperphagia-hibernation and hibernation-hypophagia were therefore missing. Ware and collaborators [40] whose captive bears showed higher (serum) cortisol during hyperphagia, nevertheless failed in confirming the winter increase. In that study, however, authors interpreted winter cortisol levels as consequence of the use of anesthetics for serum draw.

In our study, two likely concurrent explanations could be hypothesized. Despite food provisioning by keepers was continuous and bears could potentially eat non-stop throughout winter (differently from their wild or captive induced hibernating counterparts) appropriate timing, quality and amount of food were likely not made available as suggested by: a) the expression of higher appetitive behavior during hyperphagia (Jul-Sept), also directed to visitors ‐ readily responding as food providers ‐ which might describe bears as needing even more food during a crucial phase (interplay between higher FCM and appetitive feeding behavior levels, see [43]) and (b) bears’ decreasing activity and feeding behaviors (Sept-Nov), during the concurrent winter drop of both photoperiod and visitors’ provided high caloric-high nutritional peanuts (Sept-Nov). In sum, while cause-effect relation cannot be sorted out, by combining FCM and BCS results we can suppose that bears, despite the hyperphagia and fall transition increase in body mass, might have not gained enough mass before winter months which, in turn, could have not triggered the expected FCM increase [80]. In fact, our bears only showed a 11% increase of body condition score from early summer to autumn and a loss of 6% from autumn to following spring, which is much lower than the 22% increase and the 18% loss found in southern European wild brown bears [7]. The absence of data for December and January, however, cannot exclude an increase of FCM levels during that time frame. In conclusion, activity/inactivity levels, pre-denning, appetitive feeding and feeding behaviors, FCM concentration, and BCS of our captive bears changed over the seasons in a fashion (though not in the strength) similar to wild hibernating brown bears, despite a non-seasonally focused management. Based on initial results about the apparently neutral visitors’ effect on captive bears’ stress hormones [56] coupled with an overall consistent scenario described by our multifactorial analysis, we believe it is unlikely that glucocorticoid levels in hyperphagia might indicate an enhanced stress due to the visitors’ presence. At this time, however and again, we are unable to decouple a potential seasonal effect of visitors on FCM levels from the seasonal effects of the other independent environmental variables.

Wild bear hibernation response has proved to retain a certain degree of flexibility [4, 12, 18, 47–49] in response to extremely variable environmental conditions experienced in different parts of their wide geographical distribution (ranging from 20°N to the Arctic Ocean [81]). Brown bear, in fact, has been defined as a shallow hibernator species [24, 82, 83] as opposed to obligate hibernators (e.g., chipmunks, ground squirrel and groundhog [24, 84]). In the latter, for example, body temperature reaches values close to freezing, in comparison to the about 31–32°C reported for bears [4], although mechanisms employed by small and large heterotherms, functional to metabolic rate reduction, are likely to be different due to different surface-to-volume ratios and related energetic challenges [4, 85]. In some cases, in fact, hibernation still allows bears to move if necessary, and to even give birth [83]. Adaptive flexible behavioral response is likely the result of an existent innate circadian timing system synchronized with multiple proximal environmental cues, namely photoperiod (assisted by air temperature) and food availability, allowing bear to adjust their rhythms to a changing environment [12]. This dual sensitivity and flexibility associated to an innate mechanism (natural predisposition to hibernate), may explain our bear semi-hibernation response to somehow conflicting environmental cues: on one side seasonal changes in the photoperiod driving a seasonal pattern of behavior, hormones and body mass, whereas on the other side the inappropriate timing, amount and quality of food availability impeding a typical winter increase in body mass in turn likely hindering a winter increase in glucocorticoids and a complete dormancy (zero activity). Inappropriate body mass management might impair reproduction in captivity since quality and abundance of nutritional resources are crucial to positively affect body, fat, lean masses, and caloric content of hibernating brown bears [48] which, in turn, support reproductive costs right during hibernation (e.g., fetal and neonatal growth, see [86]).

Our questions in term of brown bear captive welfare derive from the following logic. Given that a hibernating bear would need proper quality and abundance of nutritional resources together with a denning place and nesting material when needed, and considering the behavioral flexibility reported in wild brown bear depending on environmental conditions, what would a proper approach be as to management of zoo-housed bears at temperate latitudes? Three options are at stake, already mentioned in the introduction: 1) not indulge on bear behavior and potential needs, and keep an unvaried management year-round (our case study) (i.e., relying on the bear natural behavioral flexibility); or (2) accommodating bears’ needs when manifested, by providing seasonal shifts in resource kinds and availability (i.e., relying on a tailored management for natural behaviors to be expressed when needed); or (3) artificially mimicking natural environmental changes (i.e., relying on the hibernation as an innate response and inducing it). Keeping in mind that variation in climate, depending on zoo location, may require different managerial approaches based on whether the bears enter an inactive state or not, we would support the second option, even if, in order to assess which approaches maximize welfare, further studies and a larger sample using all behavioral, hormonal and morphological variables would be required.

## Supporting information

S1 Table Post hoc Tukey’s test results of inactivity between months. (CSV)

S2 Table Post hoc Tukey’s test results of activity between months. (CSV)

S3 Table Post hoc Dunn’s test results of visitor numbers between months. (CSV)

S4 Table Post hoc Tukey’s test results of feeding behaviors between months. (CSV)

S5 Table Post hoc Tukey’s test results of feeding behaviors between the four phases. (CSV)

S6 Table Post hoc Tukey’s test results of body condition score (BCS) between half-months. (CSV)

S7 Table Environmental factors, behaviors, FCM and BCS during phases. (CSV)

S8 Table Environmental factors and behaviors during months. (CSV)

S9 Table BCS during half-months. (CSV)

S1 Fig Scatter plots of temperature and photoperiod values throughout the study period. (CSV)
